# Social capital and active membership in the Ghana National Health Insurance Scheme - a mixed method study

**DOI:** 10.1186/s12939-015-0239-y

**Published:** 2015-11-02

**Authors:** Christine J. Fenenga, Edward Nketiah-Amponsah, Alice Ogink, Daniel K. Arhinful, Wouter Poortinga, Inge Hutter

**Affiliations:** University of Groningen, Groningen, The Netherlands; Amsterdam Institute for Global Health and Development, P.O. Box 22700, 1100 DE Amsterdam, The Netherlands; University of Ghana, Accra, Ghana; PharmAccess Foundation, Amsterdam, The Netherlands; Cardiff University, Wales, UK; International Institute for Social Studies, The Hague, The Netherlands

**Keywords:** Social capital, Clients, Health insurance, Active membership, Ghana

## Abstract

**Background:**

People’s decision to enroll in a health insurance scheme is determined by socio-cultural and socio-economic factors. On request of the National health Insurance Authority (NHIA) in Ghana, our study explores the influence of social relationships on people’s perceptions, behavior and decision making to enroll in the National Health Insurance Scheme. This social scheme, initiated in 2003, aims to realize accessible quality healthcare services for the entire population of Ghana. We look at relationships of trust and reciprocity between individuals in the communities (so called horizontal social capital) and between individuals and formal health institutions (called vertical social capital) in order to determine whether these two forms of social capital inhibit or facilitate enrolment of clients in the scheme. Results can support the NHIA in exploiting social capital to reach their objective and strengthen their policy and practice.

**Method:**

We conducted 20 individual- and seven key-informant interviews, 22 focus group discussions, two stakeholder meetings and a household survey, using a random sample of 1903 households from the catchment area of 64 primary healthcare facilities. The study took place in Greater Accra Region and Western Regions in Ghana between June 2011 and March 2012.

**Results:**

While social developments and increased heterogeneity seem to reduce community solidarity in Ghana, social networks remain common in Ghana and are valued for their multiple benefits (i.e. reciprocal trust and support, information sharing, motivation, risk sharing). Trusting relations with healthcare and insurance providers are, according healthcare clients, based on providers’ clear communication, attitude, devotion, encouragement and reliability of services. Active membership of the NHIS is positive associated with community trust, trust in healthcare providers and trust in the NHIS (*p*-values are .009, .000 and .000 respectively).

**Conclusion:**

Social capital can motivate clients to enroll in health insurance. Fostering social capital through improving information provision to communities and engaging community groups in health care and NHIS services can facilitate peoples’ trust in these institutions and their active participation in the scheme.

## Background

Many people in sub-Saharan countries have poor access to quality health care and spend a substantial amount of their income on health care [44, 45]. This easily leads to the rural poor and vulnerable people falling into a poverty trap [38].

**Fig. 1:**
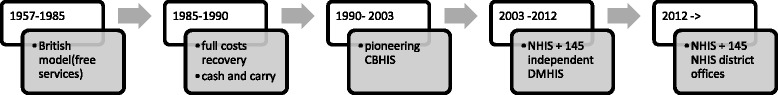
Health Financing Reforms Ghana presents the different phases of development of Ghana’s health system from 1957 till today

Upon its independence in 1957, Ghana adopted a healthcare service system that was modeled on the British system (Fig. 1). Healthcare services provided by publicly owned facilities were initially free at the point of use. This approach proved to be unsustainable as the country’s economic performance declined. In 1985, nominal fees were introduced in the form of co-payments to prevent the disintegration of the publicly funded services followed by the ‘cash and carry system’ in 1992 [3]. The introduction of user fees however, led to low utilization of healthcare facilities among the poor. In the early 1990s, voluntary community-based health insurance schemes were pioneered with the help of international donors and agencies. These risk-pooling programs provided access and financial protection for those excluded by formal schemes and user charges. The schemes were only established in some areas in the country and therefore did not ensure universal coverage.

In 2003, Ghana moved to a nation-wide implementation of District Mutual Health Insurance Schemes (DMHIS) under a National Health Insurance Scheme (NHIS) policy framework [3, 11]. Later, in 2012, the NHIS Authority reviewed the legal frame work that governed the operations of the NHIS to resolve some structural and administrative bottlenecks. The new law consolidated all 145 District Mutual Health Insurance Schemes into one NHIS. Certain functions such as claims handling were centralized in order to decrease efficiency challenges and to allow DMHIS staff to focus on awareness raising, public relations and client enrollment.

Ghana NHIS aimed to abolish user fees and improve access to health care for all residents of Ghana, allowing registered card-bearing members to access health services from both public and private healthcare providers throughout the country. The NHIS policy framework mandated the District Mutual Health Insurance Schemes to charge an annual premium between 7.2 and 48 Ghana Cedis (one GHC = 0.28 USD) per adult. Formal sector workers are exempted from paying premium because 2.5 % of their monthly salary is transferred by their employers to the NHIS fund. In order to promote access to health care amongst certain vulnerable groups, some people (the very poor; children under 18 years of age; people above 70 years of age; all pregnant women) are exempted from paying insurance premium. The NHIS covers about 95 % of Ghana’s disease burden reported at the out-patient departments [32]. To guarantee quality healthcare services, the NHIS introduced quality standards and certification of healthcare facilities in 2010 in addition to regular medical audit visits to the facilities.

The National Health Insurance Authority (NHIA) has made major progress in developing and implementing the NHIS, attaining a cumulative enrollment rate of over 70 % (close to 17 million people) in 2011. Nevertheless, at that year only 34 % of the population were active member through payment of their annual premium, suggesting that many were dropping out [33]. This mend that a majority of the population was excluded from the NHIS, while non-members continued paying out of their own pockets [29]. To investigate the NHIS operations in Ghana, a number of studies were conducted [4, 10, 24, 26, 30, 32, 43]. These studies revealed a variety of challenges to successful implementation, such as insufficient information and communication, low perceived quality of health care services, favoritism for those uninsured such as shorter waiting times at the healthcare facilities, and delays in production and distribution of insurance cards.

At the request of the NHIS Authority our study explored clients’ motives to seek healthcare and NHIS services, with the specific interest on how the socio cultural context and social relationships shape peoples’ perceptions, behavior and decision making. The findings were to support the NHIS policy and practice.

**Fig. 2:**
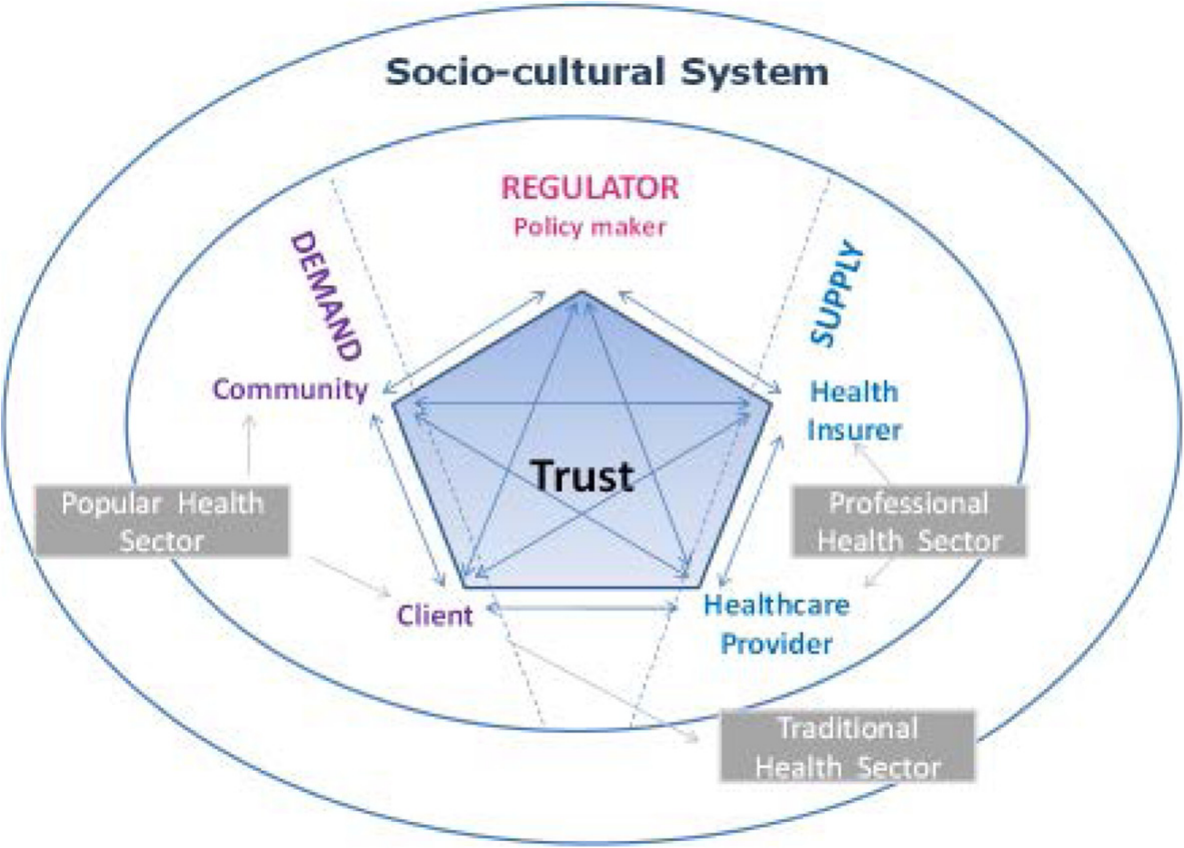
The Integrated Health Model (IHM) is the conceptual framework the researcher developed and used. This model adopts elements from different theories. Each of these theories point at the importance of socio-cultural context in people’s behavior: the socio-anthropological model of healthcare systems [28], the sociological model [6, 16, 18, 19, 37], with a specific focus on the social capital theory [46]. The IHM presents a conceptual model of healthcare systems and social relationships that influence people’s perceptions on illness and health care and health insurance services and their decision to use services

For our study we designed the Integrated Health Model (IHM), a deductive conceptual framework with inductive elements from the interviews with clients (Fig. 2).

This model adopted elements from different theories. Each of these theories point at the importance of socio-cultural context in people’s behavior: the socio-anthropological model of healthcare systems [28], the sociological model [6, 16, 18, 19, 37], with a specific focus on the social capital theory [46]. The IHM presents a conceptual model of healthcare systems and social relationships that influence people’s perceptions on illness and health care and health insurance services and their decision to use services. It reflects the daunting complexity, with potential conflicting interests and goals of stakeholders (i.e. clients, healthcare providers, health insurer) in the system, often based on asymmetric information. According North [34] high levels of uncertainty can lead to lack of trust, high transaction costs and unfavorable behavioral effects [34].

The concept of social capital is described by different authors, all referring to social connections or social networks as an important element [5, 6, 17]. Putnam et al. [37]) defines social capital as ‘catalyst of cooperation and coordination that can achieve improved social and economic outcomes’. Alesina and Ferrara [2] find that ‘social capital can reduce transaction costs and incomplete or asymmetric information in the absence of formal contracts’. Important features of social capital are trust and reciprocity [3, 5, 7, 17, 37]. In communities with high levels of social capital, people share values and information, prevent opportunistic behavior and foster reciprocal support [1, 7, 21].

**Fig. 3:**
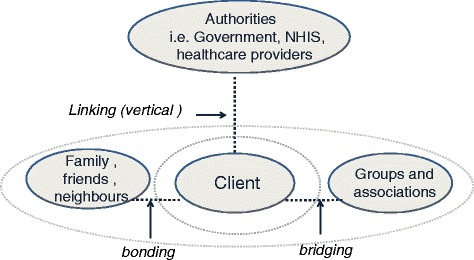
The social capital relationships shows schematically the different types of relationships (*horizontal* and *vertical*) that are subject of this study. The researcher developed this figure is on the basis of the literature study on this topic

To operationalize social capital, we can differentiate social capital according to levels of interaction namely: horizontal (*bonding and bridging*) and vertical (*linking*) relationships as shown in Fig. 3 [27, 35, 36, 39]. *Bonding* refers to social support provided by indigenous social institutions i.e. sick and vulnerable people are helped and protected by their families. Bonding is characterized by a strong feeling of solidarity, reciprocity and joined values [3].

*Bridging* social capital refers to connections across different ethnic, religious or occupational groups, yet are more or less equal in terms of their status and power [9, 25, 40]. Examples of bridging are existing support and welfare groups providing mutual support in the community. The underlying motive for this support is reciprocity. By being part of a solidarity group and securing the interest of the group, the individual also protects his or her own interests [3].

In our study we explored whether horizontal social capital is part of the Ghanaian community and if so, whether this form of social capital motivates people to actively participate in the NHIS.

*Linking* social capital is the third form of social capital, which refers to relations between communities and formal or institutionalized power or authorities in society [40]. This is what we call vertical social capital (Fig. 3).

In our study we explored whether vertical social capital motivates people to actively participate in the NHIS. We studied the relations between the (potential) clients and the healthcare provider and the NHIS.

We studied the influence of social capital on people’s motives to enroll in the NHIS. Do trusting, reciprocal relations between clients in the community (horizontal social capital) and between clients and the service providers (vertical social capital) make a difference? Earlier studies in other countries have looked at the association between horizontal social capital and health status and life expectancy [27, 31, 36, 39, 41] and access to healthcare [21] and found a positive relationship. Studies of Zhang et al. [47], Donfouet et al. [12] and Hsiao [23] explored the effect of horizontal social capital on the demand for community based health insurance (CBHI) and found higher levels of social capital associated with a higher demand for CBHI. Our study is unique in that it focusses on horizontal and vertical social capital in a national health insurance setting. We hypothesized that social capital is part of the Ghanaian society (hypothesis 1); secondly we hypothesized that horizontal and vertical social capital motivate people to active membership in the NHIS (hypothesis 2).

## Data and methods

### Study design and data collection methods

This study, conducted in 2011–2012 among NHIS insured and non-insured clients of primary healthcare facilities, took place in two regions: the predominantly urban region Greater Accra, with many people employed in the formal sector, and the rural Western Region featured by people employed in the informal sector such as in agriculture and fishery. Distances to health facilities in Western Region are higher, while in the capital region considerable choice of healthcare service is offered. In 2011, active membership in Greater Accra and Western region was 25.6 and 32.2 % respectively. Ethical clearance was obtained from Ghana Health Services. Each client was told about the study objectives and informed about the possibility to withdraw at any stage of the discussions without it having any negative consequences.

The study used a combination of qualitative and quantitative methods. This allowed method and data triangulation [20], which generally leads to more valid results and evidence building [20, 22, 42].

### Qualitative methods and data management

The application of first qualitative methods allowed generating rich data, insights in people’s perceptions on illness, health seeking behavior, community relations and relationships with healthcare providers and the NHIS. Individual interviews typically offered emic insights while the focus group interaction between participants provided insights in common issues and feelings. This deeper understanding of people’s socio-cultural views and decision-making helped in the design of survey question and interpreting the quantitative data [14, 15]. Local research staff was trained and supervised and all interviews were conducted in the local language of that particular region.

The qualitative data were collected through 20 individual interviews, 22 focus group discussions with clients and seven key informant interviews, conducted in the catchment area of the selected healthcare facilities. Topic guides were developed based on the Integrated Health Model and underlying theories and pilot-tested, while in the course of the qualitative data collection, inductive topics were added. These topics focused on cohesion, reciprocity and trust relations in the community; relations with the healthcare provider and NHIS, perceptions of services, information and communication, trust in governance of services. Participants were recruited with assistance of the local community workers and included women and men above 18 years of age, insured and uninsured. The focus group compositions consisted of different combinations such as insured only, uninsured only, mixed insurance status, mixed gender. Interviews continued until no new information transpired. Recorded interviews were transcribed verbatim, translated in English with typical local terms unchanged. Data was analyzed using the broad principle of grounded theory [22] and with support of N-Vivo-9. The coding was conducted by two people, with a third person helping where no consensus could be reached between the two people. The results were discussed and validated in two separate stakeholder workshops. During these validation meetings no missing points transpired.

### Quantitative method and data management

For the quantitative data collection, the study used a multistage sampling strategy, whereby first the 64 primary healthcare facilities were selected on the basis of their ownership (public/private), location (rural/urban) and NHIS accreditation quality scores. A second sampling stage was conducted to collect data from 1903 randomly selected households in the two regions. Thirty households were randomly sampled from within a radius of 10 km around each of the 64 selected primary healthcare facility. In total, data on 7097 individuals, aged 18 year and older and dwelling in the sampled households was collected.

During the survey respondents were asked whether they were currently enrolled or had been enrolled since the launch of the NHIS. The response options were: ‘currently enrolled’, ‘never enrolled’, and ‘previously enrolled’. To confirm active NHIS membership people had to show their valid insurance card.

The survey was designed based on the qualitative data, resulting in well-grounded questions. The survey included the following sections: identification sheet (13 questions); household demographics (six questions); social capital 45 questions including questions on trust, collective action, information and communication, social cohesion and inclusion, empowerment); employment status (seven questions); health status and healthcare utilization (51 questions); NHIS enrolment (31 questions); perceived quality of healthcare services (15 questions); perceived quality of health insurance services (35 questions); socio-cultural schemas and health seeking behavior (eight questions); consumption and expenditure (nine questions).

Forty surveyors were trained, nearly all of them were experienced field staff from Ghana University, having worked in similar research programs before. Their experience allowed for very constructive discussions and input during the training, in which all 220 survey question were briefly discussed. The survey was subsequently tested during an organized field trip and checked on errors. These were corrected and a final survey version developed. The survey language was English, however the surveyors spoke and used the local language during the interviews. Translation issues were discussed during the surveyor training in order to reach consensus on local words to be used consistently by all surveyors.

For analysis we used SPSS-20. In the analysis the continuous variable age was divided into four categories: 18–45, 46–55, 56–69 and 70+. These four categories are often used in medical literature to allow for differences in health needs between the groups. The education was divided into four categories, based on the education system in Ghana (in Section 3, Table 1).

**Table 1:** Socio-demographic variables of clients

N Total = 1903, N Valid =1804	Currently enrolled	Previously enrolled	Never enrolled	Total	Chi sq
	n (%)	n (%)	n (%)	n (%)	
Location					
Rural	337 (37.5)	159 (17.7)	402 (44.8)	898 (100)	0.172
Urban	342 (37.7)	189 (20.9)	375 (41.4)	906 (100)	
Sex					
Male	429 (37.2)	189 (16.4)	534 (46.4)	1152(100)	0.000
Female	250 (38.3)	159 (24.4)	243 (37.3)	652 (100)	
Age					
18–45	336 (31.5)	201 (18.8)	529 (49.7)	1067 (100)	0.000
46–55	116 (37.8)	63 (20.5)	128 (41.7)	307 (100)	
56–69	144 (48.5)	58 (19.5)	95 (32.0)	297 (100)	
70+	83 (62.4)	26 (19.5)	24 (18.0)	133 (100)	
Education level					
No education	105 (32.5)	73 (22.6)	145 (44.9)	323 (100)	0.001
Primary	324 (36.0)	180 (20.0)	396 (44.0)	900 (100)	
Secondary/Vocational	142 (38.9)	62 (17.0)	161 (44.1)	365 (100)	
Tertiary	103 (49.5)	33 (15.9)	72 (34.6)	208(100)	
Self-rated health					
Very good	287 (33.0)	179 (20.6)	405 (46.5)	871 (100)	0.000
Good	245 (37.7)	122 (18.8)	283 (43.5)	650 (100)	
Fair	87 (46.8)	34 (18.3)	65 (34.9)	186 (100)	
Bad	56 (65.9)	9 (10.6)	20 (23.5)	85 (100)	
Very bad	3 (60.0)	1 (20.0)	1 (20.0)	5 (100)	
Religion					
Christian	617 (38.6)	314 (19.7)	666 (41.7)	1597 (100)	0.002
Muslim	48 (33.3)	29 (20.1)	67 (46.5)	144 (100)	
Traditional religion	1 (20.0)	0 (0.0)	4 (80.0)	5 (100)	
None	13 (22.4)	5 (8.6)	40 (69.0)	58(100)	

All analyses were performed on valid entries

To measure horizontal social capital we used proxy questions on trust, solidarity and collective action in the community. To measure vertical social capital, we asked questions related to clients’ trust in formal institutions, i.e., the healthcare provider and the NHIS (in Section 3, Table 2). Clients’ views, as expressed in the interviews, helped contextualize the proxy-questions and phrase them in a socio-cultural sensitive way. To illustrate: One aspect emphasized by clients was the importance of the assembly men in the community, which led to the inclusion of a question on ‘trust in the assembly men’. To gauge vertical social capital, we included questions related to aspects such as being treated respectfully and with compassion by the healthcare staff. Fairness and non-discrimination in services were also indicated as important. Information provision and trustworthiness in providing an adequate services package as promised determined a client’s trust in the NHIS.

**Table 2:** Factor extraction through principal component analysis

	Rotation component matrix		Components		
	Factors	1	2	3	4
Horizontal SC *(trust)*	I trust most people in this community	.046	.799	.274	.077
	I trust my village elders	.035	.885	.162	.089
	I trust my Assembly man/women	.067	.827	.178	.128
Horizontal SC *(action)*	Most people in this community will help others when they are in need	.117	.319	.730	.022
	People in this community will contribute money to projects even if they don’t benefit themselves	.088	.134	.842	.139
	People in this community will collaborate to solve any health services related problem	.128	.193	.769	.038
Vertical SC *(HCP)*	The doctor/ med. assistant/ nurse are compassionate and very supportive	.769	.063	.024	.043
	The doctor/ med. assistant/ nurse treated me respectfully	.759	.070	.008	.087
	There are sufficient good doctors/med. assistants/ nurses	.646	-.084	.207	.073
	I received all prescribed drugs from the facility	.641	.061	.068	.041
	There are adequate consulting rooms and medical equipment	.662	-.066	.165	.059
	There is a well-organized and fair queuing system	.695	.122	.021	.088
	Health personnel treats insured patients in an equal way as non-insured	.588	.051	.011	.116
Vertical SC *(NHIS)*	The information from the NHIS is adequate	.010	.060	.035	.748
	The NHIS is trustworthy	.149	.093	.068	.768
	The services covered in the NHIS package are adequate	.202	.101	.079	.761

All social capital proxy questions were answered using a five-point Likert scale, ranging from 1 (strongly disagree) to 5 (strongly agree). To reduce the number of social capital questions to a workable number of factors for horizontal and vertical social capital, we developed a correlation matrix of variables. To identify the factors we conducted a principal component analysis with Varimax rotation and Kaizer Normalization (in Section 3 Table 2). To study the relation between social capital and enrolment status (categorically distributed dependent variable) we estimated a multinomial logistic regression model (in Section 3 Table 3). Because the estimate of a regressions can be biased if other explanatory variables are omitted, we controlling for age, sex, education, self-rated health, urban–rural location, which we included as dummies in the model. The regression in Table 3 is a multivariate analysis, which allows assessing all variables at the same time and look for co-founding effects and interaction between the variables.

**Table 3:** Multinomial logic regression of enrollment status^a^ and social capital factors (N Total =1903, N valid = 1727)

	Currently enrolled^a^			Previously enrolled		
*Variables*	*Estimate*	*Std error*	*P-value*	*Estimate*	*Std. error*	*P-value*
Horizontal Social Capital (Trust)	.152	.058	.009	.099	.069	.151
Horizontal Social Capital (Action)	-.016	.057	.781	.074	.068	.279
Vertical Social Capital (HCP)	.251	.058	.000	.038	.067	.568
Vertical Social Capital (Insurer)	.269	.058	.000	.245	.079	.000
*Sex*						
Female	.431	.127	.001	.682	.146	.000
Male	0^b^.			0^b^		
*Age*						
Age category 18–45	−1.707	.273	.000	−1.081	.330	.001
Age category 46–55	−1.376	.291	.000	-.833	.350	.017
Age category 56–69	−859	.289	.003	-.529	.352	.133
Age category 70+	0^b^			0^b^		
*Self-rated health status*						
Very good	-.263	1.188	.049	.317	1.438	.826
Good	-.247	1.187	.043	.170	1.437	.906
Fair	.070	1.195	.003	.272	1.446	.851
Bad	.706	1.212	.340	.007	1.484	.996
Very bad	0^b^			0^b^		
*Location*						
Rural	-.012	.117	.921	-.241	.139	.084
Urban	0^b^			0^b^		
*Education*						
None	−1.429	.246	.000	-.272	.296	.358
Primary	-.882	.187	.000	-.182	.245	.459
Secondary	-.593	.206	.004	-.270	.274	.324
Tertiary	0^b^			0^b^		

Note: ^a^The reference category is *never insured*. ^b^These variables are reference. The regression analysis was performed on valid entries

## Results

### Qualitative findings

This section presents the findings of the interviews and group discussions, revealing people’s perceptions in the community with regards to social capital. It demonstrates the presence of forms of horizontal and vertical social capital.

#### Community cohesion, reciprocity and trust

Interviews revealed that traditional family structures are still strong in Ghana. Family relations offer security through financial or other support in times of need. ‘*When I am ill my siblings or parents come and assist me, or bring me to the hospital, that is tradition here*’, told a man in Greater Accra. However, we observed changing trends in family bonding due to modernization and social developments related to education and economic development: ‘*Now the world has become difficult, family members are no more supporting anybody: ‘Everyone for himself, God for us all’ is the motto we have in this family’* (FGD male, uninsured, Western Region). People value connectivity between people beyond the extended family, in the form of organized groups and associations. These are increasingly important and seem to fill the gaps of weakening traditional social ties. This became clear from focus group discussions in both regions: ‘*We have many active groups in our community. Like me I belong to several groups…… of the men’s fellowship group I am the chair. If your mum is sick, we visit her….. we will give some money. Every time someone gets stuck somewhere, we unite and then all donate a little’* (FGD, male, uninsured, Greater Accra Region)*.* Participants mentioned benefits of being member of a group such as enhanced information sharing, capacity building, motivation, joint communal action and risk sharing. Strong moral and spiritual leadership is found to encourage participation and adherence, whereby church groups and funeral groups stand out as strong and reliable groups. Trust and unity in a group can easily diminish when leadership shows inappropriate management capacity or embezzles funds. Changes in social development and increased heterogeneity are likely factors to reduce the sense of community solidarity. In the Western Region where vast economic developments are resulting from the emerging oil industry we heard: ‘*At first the oneness was here. When I look at the time we grew up it was fine. Now things have changed…. maybe one has got a job and another not. Those working will laugh at those sitting, making that people don’t talk to each other’ (*Individual interview, male, insured, Western Region).

#### Trust in professional health sector

We refer to vertical social capital as the relationship between clients and the formal, professional health sector. The traditional healers, preferred by some people in the community in the case of certain illnesses, fall outside this ‘linking’ construct because they are part of the community’s informal support structure. We will refer to this relationship as horizontal social capital. Clients mentioned communication, attitude, devotion and encouragement as important elements in a trusting relationship with a formal healthcare provider. This is what people expect to receive but in practice they often find these inter-relational aspects poor. In addition they want the services of the organization to be reliable (availability of staff, equipment and drugs, consistent information). ‘*What I want them to provide me with is that, when the illness is serious like my illness…a nurse is around me, helping me on a bed and ask me what exactly is wrong with me, listen to me and then know what drugs to give me. That is what I expect a nurse or doctor to do for me’ (*FGD, female, uninsured, Western Region). We also found differences in client perceptions between public and private healthcare facilities: *‘…the government facilities have more qualified personnel. But when you have insurance, the reception is very bad….they don’t bother about you because they do not get real money out of it, they handle you very badly. So someone like me prefers to go to a private hospital to pay more but will be well catered for’ (*FGD, male, insured, Greater Accra*).* Private facilities are perceived as more trustworthy: *‘For the private one, it’s a private individual who build the facility and employs the staff. He will tell the employee how much to collect. At any time the supervision is there.. [eeh eeh] supervision is very intense compared to public hospitals. So when you go there they will whole-heartedly examine you and the machines they use to treat you with are more advanced than in the public facilities’ (FGD, female, uninsured, Western Region).* Another frequently mentioned complaint was health staff favoring uninsured people with quicker and better service. Relationships between clients and NHIS/DHIS were hampered by inconsistencies in information about the NHIS enrollment and renewal procedure. In certain communities, insured clients complained about changes in the provided benefits package. These perceived changes were noticed over time. Others mentioned differences in benefits packages from different healthcare providers. The insurance concept is well understood but trust in the services tends to decrease when clients are disappointed at the point of service. These insurance problems could be primarily caused by the healthcare provider offering poor services, which clearly illustrates the complexity of this tripod relationship. According to clients, the NHIS/DHIS should therefore conduct intensive supervision, ensuring that people receive the agreed care. Other clients mentioned that they understood providers could not offer services because of delays in reimbursement of claims by the NHIS. This NHIS issue disrupts consistent service provision by the healthcare provider and may subsequently influence clients’ trust in the healthcare provider.

#### Quantitative findings

NHIS active membership status was used as dependent variable in the quantitative analyses. Table 1 provides the socio-demographic characteristics, set against the status of enrollment.

We defined proxy questions and extracted four components. Two factors were composed of proxies related to horizontal social capital in the community (trust, solidarity and collective action) and two factors composed proxies reflecting vertical social capital (Trust in Healthcare Providers, and Trust in NHIS). All four social capital measures appeared internally consistent (Cronbach’s α = 0.844, 0.767, 0.809, and 0.670 respectively) although the last factor is slightly lower in reliability. The Kaiser-Meyer-Olkin measure of sampling adequacy (0.811) and Bartlett’s test of sphericity were applied (.000) guaranteeing sufficient collinearity to warrant the use of Principle Component Analysis. The cumulative percentage explained by variance of the four factors is 60 %. Factor one accounts for 27 %, factor 2 for 16 %, factor 3 for 9 % and factor 4 for 7 %.

In testing our hypothesis that social capital is positively associated with active membership in the NHIS, we found a statistically significant overall relationship between the combination of independent variables and the dependent variable. Our hypothesis proved valid for community trust (*p* .009), trust in the healthcare provider (*p* .000) and trust in the NHIS (*p* .000). This means that higher levels of community trust (horizontal social capital) and higher levels of trust in the formal institutions (healthcare provider and health insurer) result in higher active membership rates in the scheme (Table 3).

The relationship between the community’s solidarity and collective action (second horizontal social capital factor) and active membership status was not statistically significant. The regression analysis also demonstrates that active membership increases with age when comparing the age categories with the reference category 70+. Similarly, we show a significant correlation between active membership and health status, whereby people with poor health are more likely to enroll than those with good health. Education level shows a similar trend, with people who completed higher education more likely to enroll than those without education. When estimating the regression for those previously enrolled, we found only one social capital factor (trust in the NHIS) positively associated.

## Discussion and policy implication

The NHIA is keen to see its objective to offer social health insurance to the entire population realized. Inquiring into the challenges of enrollment forms therefore a key policy priority. Earlier studies have indicated that horizontal social capital in the community can be an important link with enrollment in the scheme [12, 13, 47]. In this paper we explored the role of (horizontal and vertical) social capital in people’s motives and their decision to participate in the NHIS. In the introduction of this paper we hypothesized we would find forms of social capital (horizontal and vertical) in Ghana (hypothesis 1). Furthermore, we expected a significant positive association between social capital (horizontal and vertical) and active membership in the health insurance scheme (hypothesis 2).

The triangulation of the qualitative and quantitative data shows consistency of results (See Table 4), demonstrating a high validity of our finding. It shows that different types of social capital exist in Ghana and that high levels of trust among people in the community and trust in the healthcare provider and insurer have a positive influence on enrollment in the insurance scheme.

**Table 4:** Triangulation qualitative and quantitative data

**Qualitative data**	**Quantitative data**	**Triangulation**
**Reasoning**		
*Expectations* *based on theory.*	*Hypothesis 1 (H1)* *: Various forms of social capital are present in Ghana. (Identification of horizontal and vertical SC factors)*	
*Iterative process engaging clients and integrating their emic perspectives on social relations, reciprocal support and trust.*	*Hypothesis 2 (H2)* *: Clients’ active membership in the NHIS is explained by social capital.*	
**Results**		
(Individual interviews, FGD)	(Household survey)	
Perceptions reveal existence of different forms of social capital.	4 factors identified: 2 horizontal, 2 vertical (see Table 2)	Consistent results: Both qualitative and quantitative methods identified different forms of social capital.
*Horizontal*: Traditionally strong family bonds. Multiple groups (youth, church, women), increasingly popular. Trust in traditional (informal) health system.	*Horizontal:*	The emic perspectives (qualitative) make findings more culturally relevant.
*Vertical:* Relationships of clients with healthcare providers and health insurer exist but are influenced by various issues.	- Trust (in community)	The identified forms of social capital in the interviews help quantify social capital (factors) in the survey
	- Solidarity & collective action (in community)	
	*Vertical:*	
	- Trust in HCP	
	- Trust in NHIS	
Clients value mutual support structures and groups for information sharing, motivation, communal action.	Multinomial logic regression of social capital factors on enrollment status found significant positive associations for three out of four factors: trust in the community (horizontal) and trust in the HCP and NHIS (both vertical) (Table 3). No association found between enrollment status and communal action.	Consistent for three out of four factors.
Traditional family support structures gradually fading due to social development and modernization. Group structures are increasingly important. Existence of many groups.	Regression of social capital factor on enrollment status ‘Previously enrolled’ showed a positive association for ‘trust in the NHIS’. All other factors showed no significant correlation.	Whereas perspectives revealed people engage in groups and social action, this shows no significant positive associations with active membership. Possible explanations: social action on solidarity/reciprocal support in the community does not focus on health; lack of interest in health issues.
General NHIS awareness among communities. Value health insurance concept (reduced financial risks when ill).		Communities value insurance concept, despite the fact they are not active members. This could explain the positive association between previous enrolled and trust in the insurance. Qualitative findings revealed clients’ reluctance to subscribe due to services not meeting their expectations. This reduces their trust in the services.
Trust in NHIS services dependent on reliable quality NHIS/DHIS and healthcare providers. Trust relations influenced by experienced challenges, i.e., attitude of staff, reliability of information and benefits package, unfavorable treatment for insured, insufficient monitoring.		

Our findings confirm the conclusions of earlier work by Zhang, Donfouet and Hsiau [13, 23, 47]: people living in communities rich on (horizontal) social capital are significantly more likely to participate in the scheme than people living in low-social capital communities.^1^

However, we see a trend of changing social cohesion in communities due to socio-economic developments and modernization. Although social groups in the community are still appreciated and valued (for mutual support, sharing information, motivating), a self-interest to benefit from membership in the formal scheme rather than only rely on community solidarity may motivate people’s decisions to subscribe to health insurance. Considering these changes, formal support systems may become increasingly important. Clients’ bounded rational decision to enroll depends on whether clients perceive membership as beneficial. A healthy, young adult will feel less urgency to enroll in insurance than someone who suffers from a chronic disease. The trustworthiness, reliability and quality of delivered services of the NHIS and contracted healthcare providers encourage clients’ decision making to enroll. Our study demonstrates that people with higher levels of trust in the services (vertical social capital) are more likely to enroll.

If both horizontal and vertical social capital facilitate enrollment, what are the implications of this and which policy recommendations can be made? How can horizontal and vertical social capital be capitalized on and fostered by policy makers and practitioners?

### Fostering horizontal social capital to facilitate vertical social capital

Firstly, potential sources of social capital in the community can be utilized and strengthened by providing community members with comprehensive information on the NHIS product, its benefits and up-to-date amendments, and health care services. This also applies to the services of the healthcare provider. Community leaders can play a facilitating role as they often enjoy the confidence of their communities and can take a leading role in the effort to *motivate people* to use services. Engaged community members can use their own social structures (horizontal social capital) and their own cultural-sensitive language to raise awareness among peers. This language is expected to connect better with the community’s Explanatory Models than the national media announcements on health insurance. This will not only *reduce* the *information asymmetry,* but also *overcome uncertainties* and *strengthen trust relations* between insurer and community (vertical social capital). Being in possession of all the facts, people will be able to make their own, informed decisions. Positive experiences with the service providers can be shared among peers in the community (horizontal social capital) and as such facilitate trust in services (vertical social capital).

### Fostering vertical social capital to facilitate horizontal social capital

Interest in utilizing services relies on trustworthiness, reliability and quality of delivered services of the NHIS and contracted healthcare providers. We found that managerial issues, in particular inter-relational quality aspects, such as attitude of staff and communications, are currently undermining trust in healthcare providers. Reliable service will also rely on other functions such as timely reimbursement of claims and the availability of qualified and motivated staff. This is confirmed by other studies that pointed at the relationship of low enrollment and poor quality of care [8, 10, 24], lacking information provision and institutional rigidity.

Whereas training programs can be used to strengthen management, human capacity and quality of services, services should also meet the needs of the community. Continuous monitoring of service that meets clients’ needs and expectations can be realized by engaging the community in this process. This can stimulate healthcare providers, the NHIS and their district scheme offices to become client/community-oriented and trustworthy. The active community role can facilitate horizontal social capital and empower the community to *influence their healthcare services* and *their own life*. Strengthening the communication and relationship between clients cum community and the healthcare providers and NHIS will *reduce the power dis-balance* and *facilitate trust. Common interest* of the insurer and the community is the healthcare provider offering the agreed service benefit package. While many clients believe the responsibility of control over service delivery lies with the NHIS, we argue that engagement of clients in monitoring services can *shift some power and responsibility to the clients* while offering the NHIS important management information on client perceptions of healthcare and health insurance services.

Our results are generalizable in Ghana. We have shown the strength of our approach and our use of the qualitative and quantitative methods to study the relation between social capital and active membership in the NHIS. The extracted factors were based on theory and perceptions of clients in the Ghanaian context. Further research is necessary to increase generalizability of our approach to study social capital in other countries. In addition, further research can be conducted into the demographic distributions of social capital to build scientific knowledge for the NHIS to develop policy and practice.

## Limitations

Our data were derived from two regions in Ghana so it is possible that different associations between social capital and health insurance enrollment may emerge in other settings. We have constructed our social capital factors on theorized constructs and qualitative data. However, these measures may be limited in capturing all elements that have important relations with active membership. Further research could help to improve the factors.

Based on our selection process we were able to look at social capital as individual property. To study possible variations between social groups or networks, one would need to treat social capital as collective property. These variations will stay unnoticed when looking at only individual effects of social capital [27, 35]. Studying social capital as a collective property, alongside the individual attributes, would improve scientific knowledge regarding the association between social capital and NHIS enrollment.

## Footnotes

1 Those studies focus on community-based health insurance schemes. These community-based initiatives might have stronger solidarizing influence than a remote based, decision- making national insurance program. At time of data collection (2011) the still autonomous District Mutual Health Insurance Schemes showed similarities with community-based health insurance schemes (i.e. autonomy, local management). Since 2013a new NHIS act passed and the District Schemes are no longer mutuals but part of the NHIS. This may influence clients’ feelings related to the scheme.
